# Phimosis in Adults: Narrative Review of the New Available Devices and the Standard Treatments

**DOI:** 10.3390/clinpract14010028

**Published:** 2024-02-18

**Authors:** Eleonora Rosato, Roberto Miano, Stefano Germani, Anastasios D. Asimakopoulos

**Affiliations:** 1 Urology Unit, AOU Policlinico Tor Vergata, Viale Oxford 81, 00133 Rome, Italy; eleonoraros92@gmail.com (E.R.); s-germani@libero.it (S.G.); 2 Department of Surgical Sciences, University of Rome Tor Vergata, Via Montpellier 1, 00133 Rome, Italy; mianor@virgilio.it

**Keywords:** phimosis, foreskin, circumcision, penis, lichen sclerosus

## Abstract

Background: Phimosis is the inability to completely retract the foreskin and expose the glans. The treatment of phimosis varies depending on the age of the patient and the severity of the disease; a great number of conservative or surgical treatments are currently available. Aim: To provide the first review summarizing the available options for the treatment of adult phimosis. Methods: A PubMed, Cochrane and Embase search for peer-reviewed studies, published between January 2001 and December 2022 was performed using the search terms “phimosis AND treatment”. Results: A total of 288 publications were initially identified through database searching. Thirty manuscripts were ultimately eligible for inclusion in this review. Conservative treatment is an option. and it includes topical steroid application and the new medical silicon tubes (Phimostop™) application for gentle prepuce dilation. Concerning the surgical approach, the gold-standard treatment is represented by circumcision in which tissue synthesis after prepuce removal can be also obtained with barbed sutures, fibrin glues or staples. Laser circumcision seems to be providing superior outcomes in terms of operative time and postoperative complication rate when compared to the traditional one. Several techniques of preputioplasty and use of in situ devices (which crush the foreskin and simultaneously create haemostasis) have been also described. These in situ devices seem feasible, safe and effective in treating phimosis while they also reduce the operative time when compared to traditional circumcision. Patient satisfaction rates, complications and impact on sexual function of the main surgical treatments are presented. Conclusion: Many conservative and surgical treatments are available for the treatment of adult phimosis. The choice of the right treatment depends on the grade of phimosis, results, complications, and cost-effectiveness.

## 1. Introduction

Phimosis is defined as the inability to completely retract the foreskin and expose the glans. This common condition can be congenital (primary, without signs of scarring) or acquired (secondary and pathological); the latter is a consequence of local inflammation (recurrent balanitis or balanoposthitis) or infections due to poor hygiene [1]. Some diseases like diabetes mellitus and lichen sclerosus (LS) could also cause phimosis [2,3]. It is mostly common in children in the first decade of life with a second peak of incidence occurring after the sixth decade of life [4].

The treatment of phimosis varies depending on the age of the patient and severity of disease. It should be adapted to the clinical and individual situation, considering the presence of local infections, cultural and religious aspects. Conservative treatment is an option both in congenital and acquired phimosis, especially if grade 0–2 [5,6].

About the surgical approach, circumcision remains the gold standard. Circumcision is considered a simple surgical procedure; however, an overall complication risk of 3.8% has been reported [7]. Bleeding, pain, urinary retention, recurrent phimosis, redundant skin, wound infection, necrosis, fistulas, iatrogenic hypospadias and epispadias, meatitis, meatal stenosis, concealed penis [8], non-satisfying cosmetic appearance, and impotence are the described complications, most of which may significantly impact on healthcare costs and on the patient’s quality of life [9,10]. Moreover, phimosis is strongly associated with invasive penile cancer, due to chronic infections [11].

In an effort to maintain the efficacy and reduce the risk of complications, other more conservative surgical techniques (dorsal incision, partial circumcision, preputioplasty) [12,13,14] and use of several in situ devices which crush the foreskin and simultaneously create haemostasis have been described.

To the best of our knowledge, this narrative review is the first paper providing an overview of all therapies, surgical techniques and devices for the treatment of phimosis in the adult setting.

## 2. Materials and Methods

We performed a PubMed, Cochrane and Embase search for peer-reviewed studies, published between January 2001 and December 2022. The following search terms were used to detect all full-text publications written in English: “phimosis AND treatment”. Two authors (ADA and ER) independently screened the titles and abstracts of each citation. The reference lists of the eligible articles were reviewed, and the “Related citations” PubMed feature was also utilized. Manuscripts were assessed according to their level of scientific evidence (Oxford Center for Evidence-based Medicine).

Cohort and case control studies as well as randomized trials were included. Case reports, review articles and abstracts not followed by the full text were excluded. Finally, studies referring on pediatric (exclusively) or female cohorts, as well as studies referring on animal models were excluded.

## 3. Results

**Figure 1:**
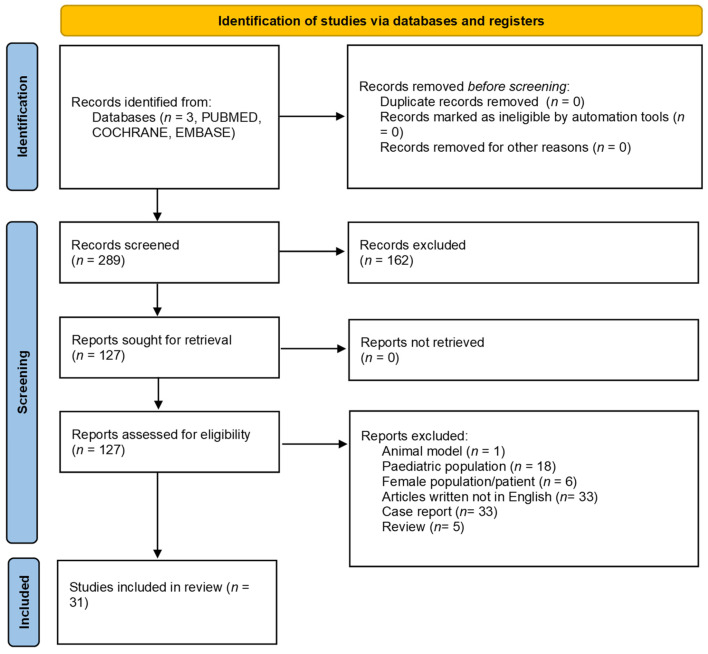
PRISMA flow chart.

A total of 288 publications were initially identified through database searching. We included studies published between January 2001 and December 2022. Thirty-one manuscripts were ultimately eligible for inclusion in this review. Figure 1 provides a diagram on the flow of information through the different phases of this systematic review according to the PRISMA criteria [15].

Fourteen studies (45.2%) were classified as case-series [4,16,17,18,19,20,21,22,23,24,25,26,27,28] and six studies (19.4%) were classified as case-comparative series, in both cases with a retrospective evaluation of the reported data [29,30,31,32,33,34]. Finally, eleven (35.5%) were prospective case–control studies [6,35,36,37,38,39,40,41,42,43,44].

### 3.1. Conservative Treatments

#### 3.1.1. Topical Corticosteroids

Non-surgical treatment of adult phimosis (Table 1) is principally based on the application of topical corticosteroids of different potency and concentration for 4–8 weeks [5]. The guidelines for the management of lichen sclerosus, an autoimmune, inflammatory dermatosis that usually leads to tightening of the foreskin in male adults and children, sometimes causing phimosis, recommend the use of a very potent corticosteroid ointment or cream (e.g., clobetasol propionate 0.05% cream or ointment) [45] (Table 2) with significant improvement in discomfort and skin tightness, reducing the need for circumcision.

Compared to placebo or manual reduction, corticosteroids significantly increase complete or partial clinical resolution of phimosis, but there is no long-term follow-up data on the durability of the results [46,47,48]. However, these drugs could induce skin atrophy (skin thinning, desirable only for the management of LS), telangiectasia (distended blood capillaries giving a spidery red spot) and immunosuppression (increasing the risk of malignancy) [45].

#### 3.1.2. The Platelet-Rich Plasma (PRP) for Lichen Sclerosus (LS)

A single retrospective study used PRP as II-line treatment of LS in 45 patients with poor outcome after long-term treatment with ultra-potent steroids (standard treatment) [22].

PRP contains several different growth factors which play a key role in the stimulation and regulation of wound healing. As described, a blood sample of 50 mL was drawn from the patient to obtain approximately 5 mL of PRP. About 2cc (range 1–3 cc) of PRP per treatment was injected in the affected areas followed by application of local antibiotic ointment. In all patients, a significant improvement in clinical conditions was observed with reduction or even disappearance of symptoms; only one patient required a circumcision procedure. Both the Investigator’s Global Assessment and the Dermatology Life Quality Index scores showed a significant difference before and after the treatment. A strict follow-up was recommended by the authors in order to detect the onset of malignant disease, although no study has documented that PRP promotes carcinogenesis [22].

#### 3.1.3. PhimoStop™

**Figure 2:**
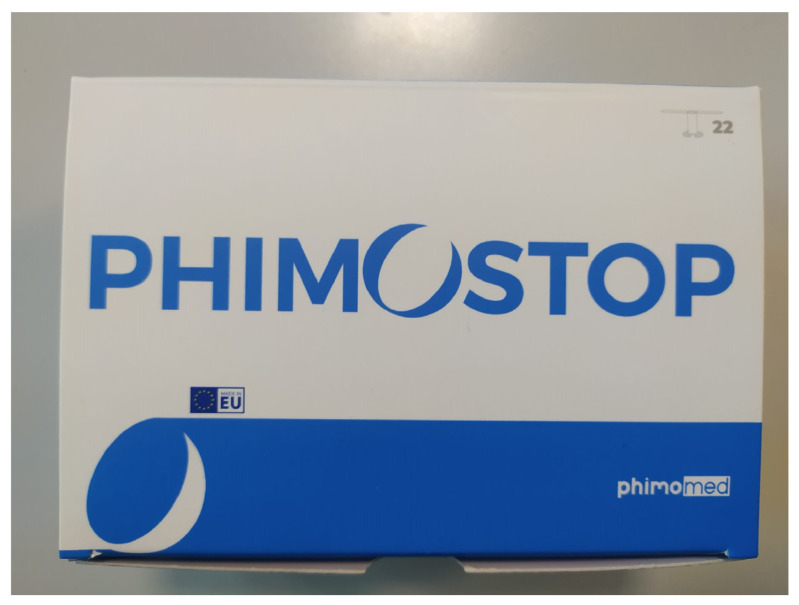

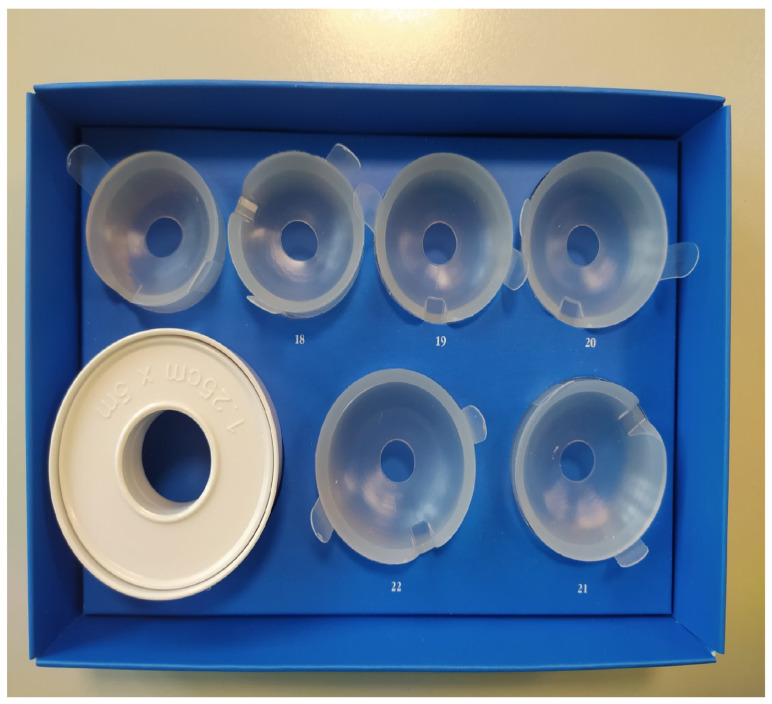

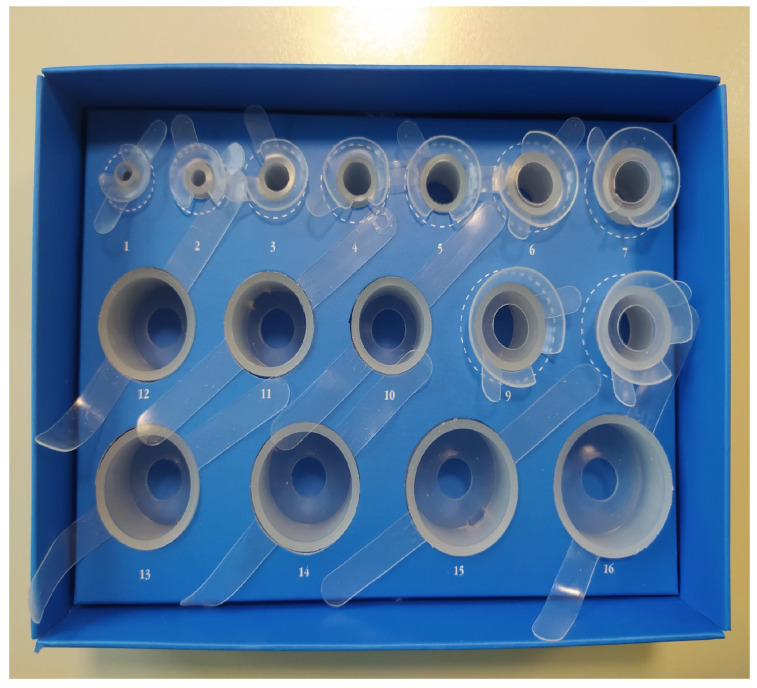
PhimoStopTM device. Standard tuboids (top row) and intermediate tuboids (bottom row). The fins of standard tuboids are fixed outside the prepuce with adhesive patches. Insertion of the intermediate tuboid on the standard one, allowing a gradual increase of the tuboid diameter of half a size.

PhimoStop™ (Phimomed S.r.l., Rome, Italy) is a certified medical device consisting of 22 medical silicone tuboids designed to apply the well-established technique of progressive and gentle skin dilation to the phimotic ring and solve phimosis without circumcision (Figure 2) [6]. The silicone tuboids are of increasing size; once the foreskin has been fully retracted, the tuboid can be applied on the glans with the central hole overlaying the urethral meatus, allowing the patient to urinate without removing the device [6]. The inner foreskin is then pulled back alongside the lateral aspect of the tuboid. Thus, the phimotic ring lies on the cylindrical portion of the tuboid undergoing a slightly forced and progressive dilation. Through the constant application of PhimoStop™, the scar ring is weakened and thinned, allowing the growth of new elastic cells that replace, in a short period of time, the inelastic ones. A single-center prospective study evaluated the effectiveness of the device in both the short- and long-term follow-up on 85 patients with acquired phimosis (grade ≤ 2 according to Kikiros) and indication for circumcision [6]. The primary outcome was to avoid circumcision in 30% of the patients [6]. The study was completed by 71/85 (83%) patients. The median duration of application of the PhimoStop™ device was 60 days. The main objective was largely achieved, since more than half (37/71, 52%) of the patients had no indication for circumcision after treatment. 31/37 (81%) patients who avoided circumcision maintained good outcomes at a median follow-up of 24 months. Finally, side effects were scarce, and in most cases, they were represented by discomfort with larger tuboid size [6].

### 3.2. Surgical Treatment

#### 3.2.1. Circumcision

Male circumcision seems to be one of the oldest surgical procedures, and it has been practiced since ancient times for medical or non-medical reasons (religious in Jewish, Muslim and traditional African cultures, social, cultural and personal reasons).

To date, it is estimated that the prevalence of circumcised men is between 12.5% and 33% of the world male population. Most of them are mainly concentrated in the USA, Canada, the Middle East and in a large portion of Africa, while in Europe, the circumcision rate is very low (around 1.5% in England) [49]. In the USA, the prevalence of circumcised men is higher among whites than blacks and Hispanics (81% vs. 65% and 54%, respectively) [17].

Apart from differences among different geographical areas, the rates of circumcision also vary according to the race, ethnicity, culture, social and economic conditions and religion. Importantly, voluntary medical male circumcision (VMMC) is a key World Health Organization (WHO) HIV preventive intervention. It is estimated that 37–39% of men worldwide are circumcised [5]. In the United States, the most common indications for adults are phimosis (52.5%), routine/ritual circumcision (28.7%), phimosis + balanitis/balanoposthitis (6.8%), balanitis (3.8%) and balanoposthitis (2.6%) [17]. Also, dyspareunia may represent a frequent indication for circumcision [19].

Circumcision is considered a simple surgical procedure; however, an overall complication risk of 3.8% has been reported [7]. The main and most frequently encountered complications of male circumcision are minor ones and are represented by wound infections, bleeding and incomplete or excessive removal of the foreskin. Meatitis and strictures of the urethral meatus are more serious and occur in 8–21% of cases, and it is believed to be due to a lesion of the frenular vessels or in the lichen sclerosus condition [49]. High-grade but rare (0.2–0.6%) complications that have been reported in the literature are as follows: urethral fistulas, necrotizing fasciitis, lymphedema, partial penile amputation and penile necrosis [49]. Mortality is very rare: 1:500,000 surgical procedures [49].

Although many reviews have been carried out, no significant differences have been obtained in terms of success and complications among the various techniques and devices used for performing circumcision [50]. Circumcision devices may be slightly preferred compared to standard surgical procedures because they reduce operative time and postoperative pain within the first 24 h [50]. However, the preferred technique should consider some contextual factors such as patient age, cost, patient preferences and values and access to trained, skilled healthcare workers and healthcare in some settings [50].

##### Techniques of Circumcision (Figure 3)

**Figure 3:**
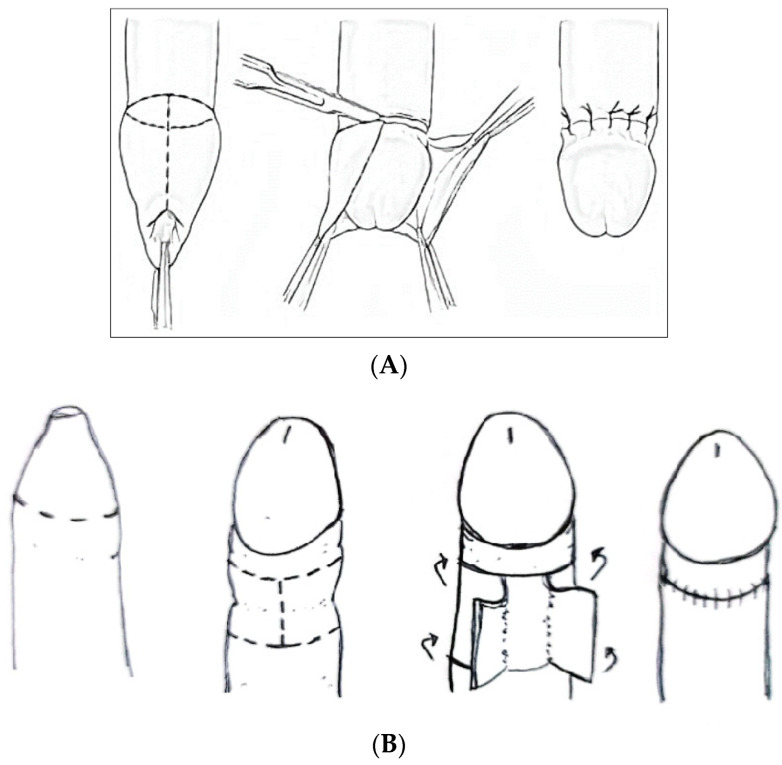
Circumcision: (**A**) Dorsal Slit; (**B**) Sleeve circumcision.

In men and older boys, the best technique seems to be sleeve circumcision (Figure 3B). In clinical practice, the incision line should be extended straight across the base of the frenulum, through the dartos fascia to the superficial lamina of the Buck fascia. After foreskin reduction, a second incision is marked, following the outline of the coronal margin and the V of the frenulum on the ventral side. The frenulum usually retracts into a V. Frenuloplasty without circumcision is not considered a gold-standard treatment, but it can be used in young patients who want to avoid or postpone total circumcision [21].

In a randomized multicenter clinical trial, Jiang ZL et al. describe a novel technique of circumcision, which retains more prepuce while sparing the frenulum. The two layers (internal and external plates) were cut off separately so that the adhering blood vessels could be stripped off [35]. Compared to the conventional dissection, the new technique provided significantly less wound healing time, scar width and recovery time, while the intraoperative bleeding volume, surgical time, and the rate of satisfaction with appearance of the penis was significantly higher. The cost of surgery to the two groups was similar [35].

Pagano C et al. proposed a dissection of the deep fascia after conventional circumcision to improve the length and circumference of the penis in a retrospective study on 36 male patients [20]. A two-step surgical procedure was described: the first step consisted of a conventional circumcision; in the second step, the skin of the penis body was retracted to the base showing ventrally the presence of the areolar tissue and Buck’s Fascia, and adherent bridles were identified and bluntly dissected. A relaxation of the adherences of the Buck’s fascia of penis and a decompression of the corpora cavernosa were obtained, causing an improvement in penile length and circumference [20].

##### Alternative Devices for Suture

The Quill™ Knotless Tissue-Closure Device (Quill™ Device)

Apart from traditional sutures for sleeve circumcision, barbed sutures, biologic glues and staplers have been used to achieve tissue synthesis after surgery. The Quill™ Knotless Tissue-Closure Device (Quill™ Device) is a unique bidirectional barb which can be fixed bidirectionally within the wound [51]. Gu C et al. performed 70 consecutive cases of sleeve circumcision by a single surgeon using the Quill™ device (3/0–4/0) via subcuticular suture to reduce suture marks on the skin of the penis. The sutures were first placed through the skin of the frenulum and the outer dorsal layer of preputial skin. Complications included one case of minor postoperative hematoma (1.4%), one case of wound infection (1.4%) and one case of pain during intercourse. All three patients (4.3%) were managed conservatively. The ultimate cosmetic results were to the satisfaction of both the patients and the surgeon [27].

DERMABOND (2-octyl cyanoacrylate from ETHICON)

The use of fibrin glue in medicine is not new, and its effect is to promote the natural clotting pathway without causing foreign body or fibrosis reactions. It has widespread use in emergency departments, particularly in children [52]. D’Arcy FT et al. report a series of 38 men circumcised using a fibrin glue, DERMABOND (2-octyl cyanoacrylate from ETHICON), after removing the outer and inner prepuce and obtaining haemostasis, the wound is approximated by using 2–5 mL of fibrin glue, handing out the glue with the tip of the applicator. Care should be taken to avoid the external urethral meatus [23]. The glue dries approximately 20 s after application [23]. All patients were satisfied with the procedure and outcome except for one patient who developed an allergic reaction, one who developed a self-limiting postoperative bleed and one who developed a focal dehiscence that required no operative intervention [23].

Circumcision staple device

Circumcision staple devices can simultaneously fulfill foreskin cutting and suturing. Disposable circumcision suture devices appeared in China in 2013, and then these have spread worldwide [27].

All devices consist of a bell-shaped glans pedestal, suture staple, ring-shaped blade, handle and shell; different sized devices are available. For phimosis patients with a small preputial ostium, the foreskin may be cut with scissors to help the inner rod insertion. The blade cuts the foreskin instantly, while simultaneously staples are placed by tightening the knob at the bottom for 3–5 s and then releasing it.

Shen J et al. compared circumcisions using two different disposable suture devices: Group A using Langhe circumcision suture devices (Jiangxi Langhe Medical Instrument Co., Ltd., Ji’an, China) and group B using Daming circumcision suture devices (Jiangsu Changshu Henry Medical Instrument Co., Ltd., Changshu, China) [33]. There are some differences between the devices’ suturing technique: the Daming device incorporates a pressure by plastic sheet upon the incision wound and the staples fix the wound outside the plastic sheet; the Langhe device directly fixes the incision wound with the staples [33]. The intraoperative blood loss of group A was higher than of group B, and two cases from group A underwent a second operation. On the other hand, group B was characterized by longer staple-shedding time after surgery, and these patients also suffered longer postoperative edema, especially on the site of frenulum of prepuce, greater postoperative pain and higher incidence of postoperative infection [33].

Lv BD et al. conducted a prospective randomized trial to assess the benefits of a new disposable circumcision suture device (DCSD, Jiangxi Yuansheng Lang He Medical Instrument Co., Ltd., Ji’an, China): 942 patients were equally divided into three groups (conventional circumcision, Shang ring and disposable suture device group) [36]. Operation time and intra-operative blood loss, and intra-operative and post-operative pain were significantly lower in the Shang ring and suture device groups compared to the conventional group. Patients in the suture device (80.57%) and Shang ring (73.57%) groups were more satisfied with penile appearances compared with the conventional circumcision group (20.06%, *p* < 0.05) [36]. The authors concluded that their modification of the traditional anesthetic and surgical methods of circumcision reduced the number of incidences of post-operative complications, intra-operative and post-operative pain and improved penile appearance and patient satisfaction [36].

Similar results were shown in a prospective non-randomized controlled study, using the same device (DCSD, Jiangxi Yuansheng Lang He Medical Instrument Co., Ltd., Ji’an, China), on 582 cases of excess foreskin and 62 phimosis patients that underwent circumcision (DCSC *n* = 295; conventional suture approach *n* = 287). Nevertheless, a multivariate logistic regression with likelihood ratio test revealed that phimosis was the significant predictor of edema occurrence postoperatively (*p* = 0.025) [37].

Han H. et al. randomized 124 adult male patients to perform novel penile circumcision suturing devices (PCSD, Changshu Henry Medical Instrument Co., Ltd., Changshu, China) or SR (Shang Ring) circumcision: there were no significant differences in blood loss (*p* = 0.054), in VAS score evaluation at the operation time, at 6 or 24 h after surgery (*p* > 0.05) or in the rates of edema, hematoma and incision dehiscence; in the PCSD group wound healing times were significantly longer (30.2 ± 4.9 vs. 15.7 ± 3.0 days, *p* < 0.01), but the cosmetic results were more satisfying (*p* < 0.01) at three weeks after the operation. The mean costs (US dollars) for the two groups were 259.6 ± 3.8 and 267.6 ± 8.4 (*p* < 0.01) [38].

Wang J et al. compared the results obtained in patients treated with sleeve circumcision or a technique using the Langhe device: no significant difference in postoperative pain, wound healing, or satisfaction were reported between the two groups for any day of follow-up (*p* > 0.05), reducing operative time and blood loss [40].

Su Q et al. compared the results of 241 male patients submitted to traditional circumcision (Group A = 79), modified circumcision (dorsal slit, Group B = 80) and disposable suturing device circumcision (TONCARE, Group C = 82). The operation time and volume of blood loss in groups B and C were significantly lower than those in group A (*p* < 0.05). Groups A and B were superior to group C in terms of the 6 h postoperative visual analog scale score and appearance satisfaction (*p* < 0.05), but there were no differences in the 7-day postoperative pain score and total healing time (*p* > 0.05). The costs in groups A and B were lower than that in group C (*p* < 0.05) [29].

##### Laser Circumcision

Aside from the conventional and device-based circumcision, laser circumcision has also been described. With the goals of complete removal of the foreskin, fine hemostasis, wound healing, cosmetics and patients’ satisfaction, laser circumcision has been tested on both pediatric and adult populations. To our knowledge, there is one prospective randomized study including a pure adult population [43] and one retrospective study [34] including both adults and children.

The prospective randomized controlled clinical study compared conventional circumcision (150 patients) to the modified CO_2_ laser circumcision technique (150 patients) [43]. There was no statistically significant difference in age distribution and indications between the two groups. Compared with the conventional group, there was shorter operative time [21.1 ± 2.7 vs. 10.5 ± 0.9, *p* < 0.05], less blood loss and a lower postoperative complication rate (mainly of postoperative pronounced oedema of the prepuce) in the laser group. The CO_2_ laser technique was associated with much less pain, as quantitated by a 10-point visual analogue scale pain score at both one day and seven days postoperatively. The only disadvantage associated with the use of the CO_2_ laser observed was the possible delay of wound healing compared with the conventional method. Wound dehiscence was observed in one patient in the laser group (vs. none in the conventional circumcision), but the patient had had sexual intercourse at 23 days postoperatively, despite having been advised to avoid sexual intercourse for six weeks.

Ronchi et al. retrospectively evaluated the medical records of 482 patients who had been circumcised under local anesthesia traditionally (168 patients-Group A) or using a CO_2_ laser (314 patients-Group B) [34]. Pain was evaluated using a verbal numerical rating scale for pain assessment. Postoperative wound swelling, bleeding, infection and pain were assessed at 4 h, 24 h and 7 days after surgery. There were no significant differences between the two groups in terms of bleeding and infections. The operating times were significantly lower in group B (23.1 ± 2.8 vs. 12.8 ± 0.9 min, *p* < 0.001). Pain scores were low, and there was less pain in Group B than in Group A during the first 4 h (1.8 vs. 3.7; *p* < 0.002) as well as at 1 day (*p* < 0.002) and 7 days (*p* < 0.001) postoperatively. The cosmetic results were superior in Group B, and significantly lower rates of buried penis were observed in Group B (10.7% vs. 2.9%, *p* < 0.002). In conclusion, the use of a CO_2_ laser was associated with a shorter operative time, less wound irritation and better cosmetic appearance compared with standard surgical techniques for circumcision [34].

#### 3.2.2. Circumcision in Elderly Patients

In this category of patients, surgery is frequently associated with anxiety related to operation time and occurrence of bleeding.

In the Mu J et al. study, 132 elderly males underwent circumcision with four different surgical methods: Group A (traditional male circumcision, *n* = 38), Group B (sleeve circumcision, *n* = 23), Group C (Shang Ring circumcision, *n* = 42) and Group D (suturing device circumcision, Jiangxi Yuanshenglang Medical Equipment Technology Co., Ltd., Yongfeng City, Jiangxi, China, *n* = 29) [32]. Group C (SR circumcision) exhibited the shortest operation time and the least blood loss, but the longest healing time. Furthermore, some patients experienced incrustation and oedema even after the SR was removed, due to the thicker foreskin in elderly males [32]. The major risk of hemorrhagic events in cases of severely thickened prepuce with enlarged veins is a contraindication for SR, and in these cases, sleeve circumcision appeared to be the best treatment. Instead, short operation and recovery times obtained by SR or stapler device reduce pain stimulation, which could cause heart burden, myocardial ischemia, severe angina pectoris and even myocardial infarction in elderly patients [32].

#### 3.2.3. Effect of Circumcision on Sexual Function

The evidence concerning the effect of circumcision on sexual function/pleasure and on sensitivity of the glans is lacking and not known. The corneum epithelium that covers the circumcised glans may lead to some degree of reduction in penile sensitivity, although there is no evidence that this event modifies the time to orgasm or the sexual satisfaction [49]. Circumcision seems to have no overall adverse effect on penile sensitivity, sexual arousal, sexual sensation, erectile function, premature ejaculation, ejaculatory latency, orgasm difficulties, sexual satisfaction, pleasure or pain during penetration [53], while in some studies, it has shown benefits on sexual function, sensation, satisfaction and pleasure for males circumcised neonatally or in adulthood [53].

Fink KS et al. examined sexual function outcomes in men who have experienced sexual intercourse in uncircumcised and circumcised conditions: of the responders, 47% reported that sex was physically more pleasurable, and 47% also said that their sex lives were more satisfying after circumcision; overall, 62% of the men were satisfied with having been circumcised [24].

Czajkowski M et al. investigated the effect of male circumcision on erectile function and satisfaction with the appearance of the genitals. The study outcomes were obtained using questionnaires such as visual analogue scale (0–10 for itching, burning, penile pain and penile pain during intercourse), International Index of Erectile Function (IIEF-5) and Male Genital Self Image Scale 7 (MGSIS-7) to assess the changes in patients’ sexual functioning [54]. Before the circumcision, 59/69 patients (86%) reported some subjective symptoms of phimosis (in order of frequency, pain during intercourse, itching and burning, penile pain) [54]. These symptoms completely disappeared at three months from surgery, and all patients achieved significant improvement in both obtaining and maintaining an erection (based on IIEF-5 score: 13.3 ± 9.5 vs. 15.4 ± 10.2, *p* < 0.001). Also, satisfaction with genital self-image increased significantly (17 ± 4.3 vs. 21.9 ± 4.2, *p* < 0.001) [54].

### 3.3. Preputial Sparing Techniques

The term preputioplasty denotes various surgical techniques directed at resolving phimosis without radical or partial circumcision. Prepuce-sparing techniques have been developed to improve the aesthetic outcomes of radical circumcision through an increased preservation of the penile foreskin [55]. Six different preputial sparing techniques have been reported for the treatment of phimosis [56], namely, the triple incision plasty, the “Heineke–Mikulicz” technique, ventral V-plasty, Y-V plasty, trident preputial plasty and Z-plasty. With the exception of the Heineke–Mikulicz and the Y-V plasty, all the other techniques have been described only in the pediatric population, and consequently their description was not included in this manuscript.

#### 3.3.1. Prepuce-Sparing Plasty and Simple Running Suture (Figure 4)

Monarca C et al. describe their five years’ experience of prepuce-sparing plasty in 52 patients that were eligible to undergo phimosis surgery [18]. The first incision is made on the external lamina of the prepuce; the second one on the internal lamina in an oblique opposite direction in order to allow the total removal of the phimotic ring and to increase the circumference of the two laminae that are then realigned and sutured with 5/0 Monocryl simple running sutures [18]. Evaluation of the results was made through comparative photos and verified by using presence/absence of recurrence, scarring evaluation and VAS for patient satisfaction; there was no pathological scarring during follow-up and there were no phimosis recurrences. Finally, all patients restarted normal sexual activity [18].

**Figure 4:**
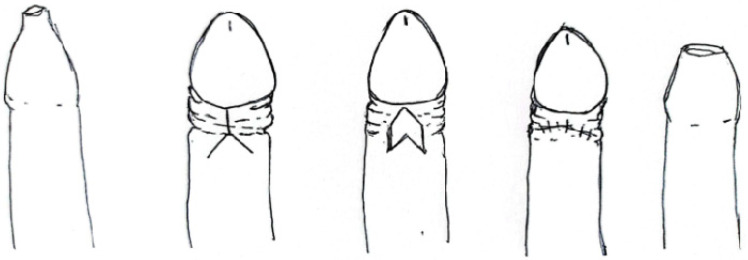
Y-V plasty.

#### 3.3.2. Y-V Preputioplasty (Figure 4)

A Y-V plasty procedure to relieve phimosis was first described by Ebbehøj’s group in 1984. This procedure is limited to men who can partially retract the foreskin [25].

In this technique, the constricting phimotic band was incised with a single, full-skin thickness dorsal cut. The incision was extended distally in 2 directions to form a “Y” shape. The resulting triangular flap was advanced proximally over the defect and sutured with a 4/0 vicryl. Munro NP et al. reported the outcomes of this technique on 89 males: 12 patients (40%) were very satisfied and 10 (33%) were satisfied, whereas 4 (13%) were indifferent and 4 (13%) were dissatisfied. Only two patients have subsequently undergone circumcision [25].

#### 3.3.3. Heineke-Mikulicz Preputioplasty (Figure 5)

Heineke-Mikulicz preputioplasty (HMP) is a foreskin-preserving surgical treatment for phimosis in the adult population. The technique is performed using a 2–3 cm vertical incision over the phimotic band on the dorsal surface to just above Buck’s fascia and an additional incision on the ventral surface if phimosis remains persistent after dorsal release. The incision is closed horizontally in 2 layers [16].

**Figure 5:**
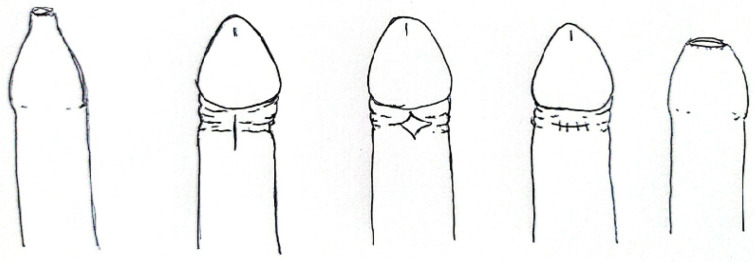
Heineke-Mikulicz Preputioplasty.

Xu AJ et al. report the data of HMP on seven patients: two patients required both dorsal and ventral incisions, no intraoperative complications were reported and one patient reported bothersome phimosis secondary to scar formation treated successfully with triamcinolone [16].

### 3.4. In Situ Devices

The first devices used to simplify circumcision were the Gomco clamp and the Plastybell devices. These ones were typically developed for infant circumcision [56]. Device-based techniques generally provide protection to the glans, and they may be safely performed by nurses and other non-physician healthcare providers [50].

Nowadays, PrePex and Shang Ring are two devices introduced to prevent a lot of sexual transmitted infections such as HPV, HSV and HIV in Africa and other countries because they permit circumcision in all conditions and in the outpatient context [31]. Contraindications for their use are penile anatomical abnormalities, chronic paraphimosis and active genital infections [57].

#### 3.4.1. The Gomco Clamp

The Gomco instrument is the leading instrument for medical neonatal circumcision in the USA. It has been approved by the US Food and Drug Administration (FDA), marketed and used since 1935 [44]. The Gomco clamp is a metal, sterilizable instrument, available in sizes from infant to adult. The clamp was applied to the penis and afterwards, the foreskin was excised with a surgical scalpel [44]. While there are very few complications from the Gomco method, mismatching of parts from different-sized instruments or different manufacturers may cause tearing of tissue [44].

Millard P et al. conducted the first randomized controlled trial on adult male patients, comparing it to other techniques. A total of 200 (83%) patients participated in the study, randomized in two groups (Gomco clamp vs. standard circumcision). After the clamp application and 5 min passed, the foreskin was excised with a surgical scalpel. The instrument was then removed, and the apposed skin-mucosal edges sealed with skin adhesive. The wound was covered with absorbent gauze [44]. There were no serious adverse events or time difference in this study [44].

#### 3.4.2. The PrePex Device (Figure 6)

The PrePex (Circ MedTech Ltd., Tel Aviv, Israel) is a device which can be placed in 3 min, under topical anesthesia [57]. The manufacturer’s cost per PrePex device for this study was USD20.00 (not including the costs of personnel, other accessories and facilities) [28].

The foreskin remains intact and is compressed by radial elastic pressure leading to distal necrosis. The necrotic skin can be removed at 5–9 days post-placement. The adverse events are rare (~1.7%), mainly due to displacement or self-removal [57]. Pain after Prepex placement was mild and was reported at 30 min. Some authors reported an unpleasant smell during the first week after PrePex placement in a great part of the patient series. In a series, wound healing was certified as complete in 56.7% (185/326) PrePex and 98.7% (74/75) dorsal slit circumcisions (*p* < 0.0001) [28].

**Figure 6:**
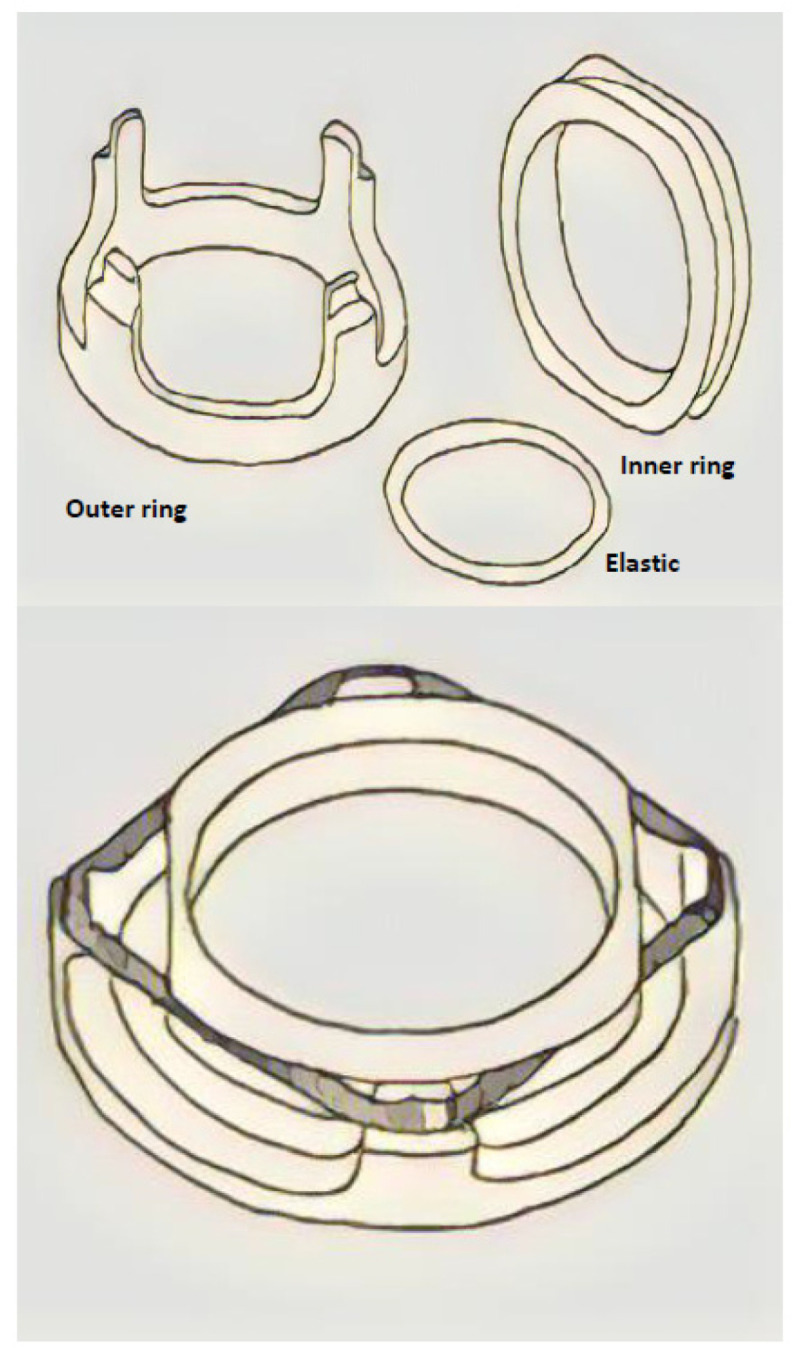
PrePex device.

#### 3.4.3. The Shang Ring™ Device (Figure 7)

The Shang Ring™ (Wuhu Shengda Medical Treatment Appliance Technology Co., Ltd., Wuhu City, Anhui, China) is a single-use device which consists of two concentric plastic rings: the inner ring and the outer ring. The inner ring is lined with a soft silicone pad, which provides a smooth and non-bioreactive surface against the surgical wound. The outer ring consists of two halves which are hinged together at the same end. On each side of the halves, there is a locking clasp which allows for locking itself with inner ring [31].

**Figure 7:**
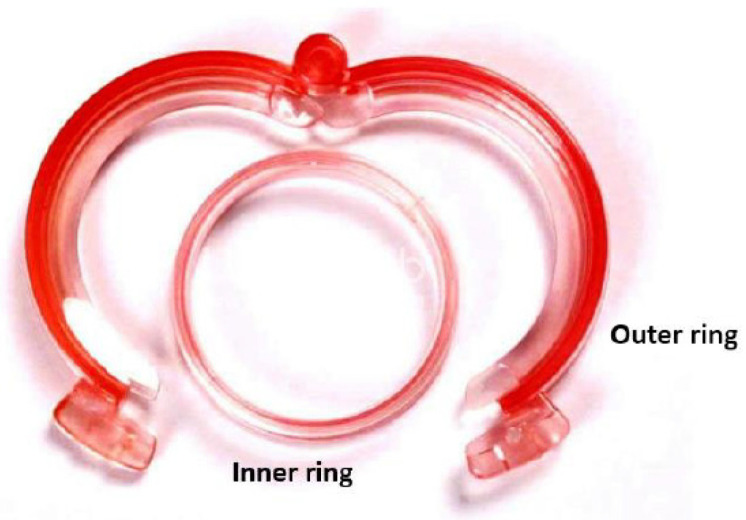
The Shang Ring™ device.

When performing circumcision, foreskin will be everted and locked between the inner ring and outer ring. Thus, a sandwich including the inner ring, redundant foreskin and outer ring will be formed [31]. Essentially, the rings act as a clamp against the redundant foreskin before it is removed by surgical scissors.

After 7–10 days, the Shang Ring can be removed as follows. First, the outer ring is unlocked and removed by a specially designed Shang Ring removal tool. Then, the inner ring is separated from the wound margin, and it should be cut using a blunt-end scissor. Local anesthesia is required during the procedure.

Wu X. et al. report a large case-series including 702 adult patients (ages ranged 18–65 years old) treated with the SR [31]. The average operative time of the adult group was 4.26 ± 2.57 min, and the timing of ring removal was 11.59 ± 3.28 days (literature time reported is 7–9 after surgery). The most common complications are infections (in 8 patients, 1.14%), postoperative bleeding (in 2 patients, 0.3%) caused by fall of ring or mild pain (48 h after surgery) in all patients during nocturnal erections. In a percentage of 30.06% of patients, yellow/white tissue exudate/lymph exudate occurred. Mild edema usually disappeared about two weeks after surgery, and severe edema did not disappear until 2–3 months later. In a few cases, the edema persisted after half a year [31].

Everting the foreskin has been reported to be the most difficult step; a modification, known as the no-flip technique, eliminates the need to evert the foreskin and simplifies device removal. With this technique, the inner ring is proximal to the wound, and it is necessary to cut off the inner ring as the ring cannot be safely slipped over the wound and off the penis [26]. Barone MA et al. conducted a randomized controlled trial of no-flip SR circumcision with removal seven days post circumcision versus spontaneous detachment [26]. The median time to detachment was 14.0 (IQR: 7–21, range: 5–35) days. The no-flip technique and spontaneous detachment are safe, effective and acceptable [26].

Lei HJ et al. conducted a single-center prospective study to compare the clinical effectiveness and safety of adult male circumcision using the SR with the no-flip technique versus the Dorsal Slit (DS) surgical method [39]. The no-flip SR method was found to be superior to the DS method for its short operation time (<5 min, *p* < 0.001), involving less pain (during the procedure and >24 h, *p* < 0.001), bleeding (*p* = 0.001), infection (*p* = 0.034) and resulting in a satisfactory appearance (*p* < 0.001) [39].

## 4. Conclusions

Conservative treatment for adult phimosis is an option, and it includes the application of topical steroids or the very promising Phimostop™ device. The gold-standard surgical treatment is represented by circumcision in which barbed sutures, fibrin glues or staples could be used after prepuce removal for tissue synthesis. Laser circumcision seems to provide superior outcomes in terms of operative time and postoperative complication rate when compared to the traditional one. Preputioplasty could be proposed in selective cases for aesthetic reasons, while some in situ devices have been described. In elderly patients, SR or stapler devices should be preferred for short operation and recovery times and minor pain stimulation (which could cause heart burden and myocardial ischemia).

The choice of the right treatment depends on the grade of phimosis, results, complications, surgeon’s preference and cost-effectiveness.

## Figures and Tables

**Table 1:** Summary of conservative treatments for adult phimosis [6,22,46].

Treatment	Advantage/Disadvantage	Side Effects
Phimostop™ [6]	Shaped silicone tuboids of increasing size to obtain a non-forced dilation of the prepuce	Scarce: discomfort with larger tuboid size
Platelet-rich plasma (PRP) [22]	Reduction/disappearance of symptoms and/or of lichen sclerosus	Risk of malignant disease (actually no study demonstrates that PRP promotes hyperplasia, carcinogenesis, or tumor growth)
Topical corticosteroids [47]	Complete or partial clinical resolution of phimosis (long-term follow-up not available)	These drugs could induce skin atrophy, telangiectasia and immunosuppression (increasing the risk of malignancy)

**Table 2:** Potency of main topical corticosteroids (British National Formulary) [45].

Potency	Topical Corticosteroids
Very potent	Clobetasol propionate 0.05% Diflucortolone valerate 0.3%
Potent	Bethametasone dipropionate 0.05% to 0.064% Bethametasone valerate 0.1% to 0.12% Diflucortolone valerato 0.1% Hydrocortisone butyrate 0.1% Mometasone furoate 0.1% Triamcinolone acetonide 0.1%
Moderate	Betamethasone valerate 0.025% Clobetasone butyrate 0.05%
Mild	Hydrocortisone 0.1% to 2.5%

## Data Availability

The datasets used and analyzed for the current review are available from the corresponding author upon reasonable request.
